# An Oral Vaccine Based on U-Omp19 Induces Protection against *B. abortus* Mucosal Challenge by Inducing an Adaptive IL-17 Immune Response in Mice

**DOI:** 10.1371/journal.pone.0016203

**Published:** 2011-01-14

**Authors:** Karina A. Pasquevich, Andrés E. Ibañez, Lorena M. Coria, Clara García Samartino, Silvia M. Estein, Astrid Zwerdling, Paula Barrionuevo, Fernanda S. Oliveira, Christine Seither, Heribert Warzecha, Sergio C. Oliveira, Guillermo H. Giambartolomei, Juliana Cassataro

**Affiliations:** 1 Laboratorio de Inmunogenética, Hospital de Clínicas “José de San Martín,” Facultad de Medicina, Universidad de Buenos Aires (UBA), Buenos Aires, Argentina; 2 Facultad de Farmacia y Bioquímica, Instituto de Estudios de la Inmunidad Humoral (IDEHU-CONICET), Universidad de Buenos Aires (UBA), Buenos Aires, Argentina; 3 Laboratorio de Inmunología, Departamento de Sanidad Animal y Medicina Preventiva, Facultad de Ciencias Veterinarias, Universidad Nacional del Centro de la Provincia de Buenos Aires, Tandil, Argentina; 4 Department of Biochemistry and Immunology, Institute of Biological Sciences, Federal University of Minas Gerais, Belo Horizonte-Minas Gerais, Brazil; 5 Department of Biology, Technische Universität Darmstadt, Darmstadt, Germany; Federal University of São Paulo, Brazil

## Abstract

As *Brucella* infections occur mainly through mucosal surfaces, the development of mucosal administered vaccines could be radical for the control of brucellosis. In this work we evaluated the potential of *Brucella abortus* 19 kDa outer membrane protein (U-Omp19) as an edible subunit vaccine against brucellosis. We investigated the protective immune response elicited against oral *B. abortus* infection after vaccination of mice with leaves from transgenic plants expressing U-Omp19; or with plant-made or *E. coli*-made purified U-Omp19. All tested U-Omp19 formulations induced protection against *Brucella* when orally administered without the need of adjuvants. U-Omp19 also induced protection against a systemic challenge when parenterally administered. This built-in adjuvant ability of U-Omp19 was independent of TLR4 and could be explained at least in part by its capability to activate dendritic cells *in vivo*. While unadjuvanted U-Omp19 intraperitoneally administered induced a specific Th1 response, following U-Omp19 oral delivery a mixed specific Th1-Th17 response was induced. Depletion of CD4^+^ T cells in mice orally vaccinated with U-Omp19 resulted in a loss of the elicited protection, indicating that this cell type mediates immune protection. The role of IL-17 against *Brucella* infection has never been explored. In this study, we determined that if IL-17A was neutralized *in vivo* during the challenge period, the mucosal U-Omp19 vaccine did not confer mucosal protection. On the contrary, IL-17A neutralization during the infection did not influence at all the subsistence and growth of this bacterium in PBS-immunized mice. All together, our results indicate that an oral unadjuvanted vaccine based on U-Omp19 induces protection against a mucosal challenge with *Brucella abortus* by inducing an adaptive IL-17 immune response. They also indicate different and important new aspects i) IL-17 does not contribute to reduce the bacterial burden in non vaccinated mice and ii) IL-17 plays a central role in vaccine mediated anti-*Brucella* mucosal immunity.

## Introduction

Mucosal surfaces (e.g. gastrointestinal, respiratory and urogenital tracts) are the initial sites of contact and entry for a vast majority of pathogens; hence the induction of protective immunity at these mucosal surfaces is usually an expected attribute in the field of development of new vaccines [1]. Currently licensed human or animal vaccines are generally administered by the parenteral route; nevertheless parenterally-administered vaccines are poor inducers of mucosal immune responses [1]. Alternatively, mucosal-administered vaccines have the potential ability to induce humoral and cell-mediated immune responses at mucosal sites and at the systemic level, likewise [1]. This attribute of mucosal vaccines together with their needle-less, noninvasive immunization approach make them a very attractive vaccination choice.

Among oral delivery systems, plant-based edible vaccines are endowed with all the attractive features of mucosal vaccines along with other distinctiveness unique to plant expression systems, such as the lack of requirement of fermentation and protein purification processes, the cost-effective production because of the low energy input and the low cost of supplies and the easy vaccine transportation, preservation and delivery [2]. Moreover, edible vaccines could be particularly suited for meat-markets-destined farm animals, as repeated injections can deteriorate the carcass quality [3].

Brucellosis is a world widespread zoonotic disease that is transmitted from domestic animals to humans. It is mostly caused by *Brucella abortus* and *B. melitensis* and is frequently acquired by ingestion, inhalation, or direct contact of conjunctiva or skin-lesions with infected animal products [4]. Bacteria spread from the site of entry to different organs causing the acute disease symptoms and developing localized foci of infection. There it survives intracellularly in the mononuclear phagocytic system leading to the chronic disease [5], [6]. The human disease represents an important cause of morbidity worldwide whereas animal brucellosis is associated with serious economical losses caused mainly by abortion and infertility in ruminants [4]. While a human vaccine would be valuable for individuals who may be occupationally exposed to *Brucella* or consume unpasteurized dairy products from areas in which brucellosis is endemic, human brucellosis incidence can be reduced by control of the infection in domestic animals [7]. Thus, prevention of animal infection by vaccination is a key issue [8], [9]. Currently, there is no available vaccine against human brucellosis and all commercially available animal vaccines are based on live, attenuated strains of *Brucella* (*B. abortus* S19 and *B. abortus* RB51 against bovine brucellosis and *B. abortus* Rev.1 for sheep and goats) [9]. Despite their effectiveness, these vaccines have disadvantages such as being infectious for humans, interfering with diagnosis, resulting in abortions when administered to pregnant animals and allowing the regional spread of vaccine strain [9], [10]. Thus, improved vaccines which combine safety and efficacy to all species at risk need to be designed. As the mucosal surfaces are the main sites of entry of *Brucella* to the body, the development of a mucosal-administered vaccine for brucellosis seems to be a rational option.

Throughout the last years we and others have made efforts to develop improved vaccines against brucellosis, without the above mentioned drawbacks [11]–[18]. Subunit vaccines, like recombinant proteins, are promising vaccine candidates because they are safer, well defined, not infectious and can not revert to virulent as live attenuated vaccines.

It is well established that the production of interferon-gamma (IFN-γ) by T helper (Th) 1 cells as well as CD8^+^ T cell-mediated responses are key mediators of protective immunity against *Brucella* infections, whereas Th2 responses are minor contributors in host resistance to this intracellular bacterium infection [19], [20]. Up to now, the function of Th17 cell responses in immunity to *Brucella* organisms has been scarcely studied. Nevertheless, Th17 responses have been shown to contribute to host defense against several extracellular pathogens such as *Klebsiella pneumoniae* [21], [22], *Citrobacter rodentium* [23] and *Candida albicans* [24] as well as against intracellular microorganisms such as *Listeria monocytogenes*, *Salmonella enterica* or *Mycobacterium tuberculosis* [23], [25].

The success of a subunit vaccine is strongly associated with its composition and its administration route. *B. abortus* 19 kDa outer membrane protein (Omp19) is a lipoprotein and is expressed broadly within the *Brucella* genus [26]. The expression of this protein has been shown to be crucial for the induction of a protective response by the vaccine strain *B. abortus* S19, since the abrogation of its gene in this strain leads to the loss of its protective ability in heifers [27], indicating that this protein would be a key component of a subunit vaccine against brucellosis. Furthermore, we have previously reported that recombinant Omp19 -when administered with the mucosal adjuvant cholera toxin (CT)- is a protective mucosal antigen that confers protection against an oral challenge with virulent *Brucella* [16]. The ability of Omp19 when given orally to mice with CT heightened our proposal of developing an oral vaccine against *Brucella* spp. However the use of adjuvants in oral administered vaccines involves some drawbacks: i) CT is a potent experimental adjuvant but is also toxic [28] ii) there are very few mucosal adjuvants to test and most of them are based on bacterial toxins or their subunits [28], [29]. A putative approach to avoid the need of external adjuvants consists in having the 2 factors required to elicit a protective response in the same molecule: the protective epitopes and the adjuvant activity. In the present work we evaluate if Omp19 from *Brucella* spp. -as an edible plant-made vaccine against brucellosis- would fulfill the above-mentioned requisites and, study the implicated immune mechanisms elicited upon vaccination.

## Results

### Plant-made U-Omp19 elicits a protective response against an oral *Brucella* challenge

**Figure 1:**
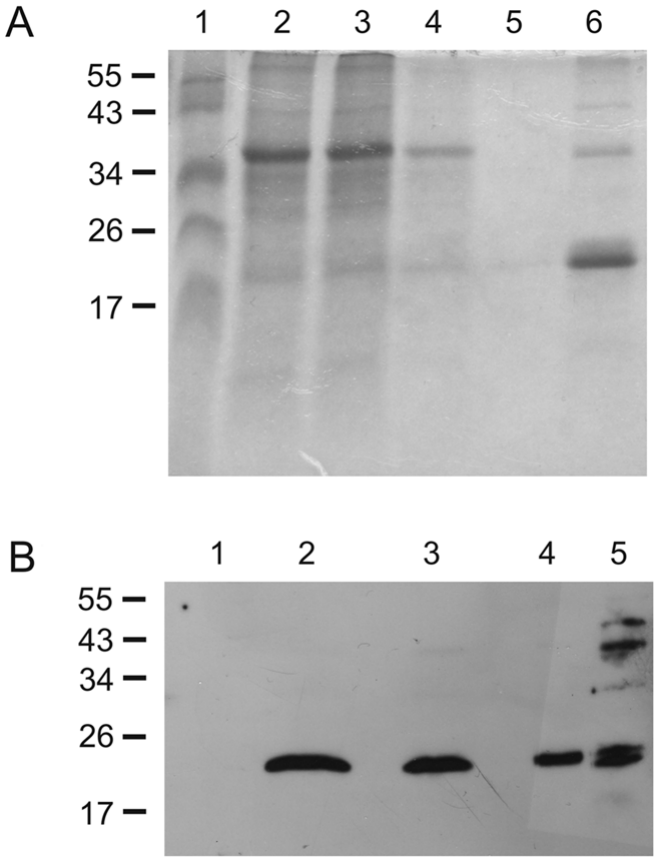
Omp19 expression in plants. (**A**) SDS PAGE of different fractions obtained from immobilized metal affinity chromatography. 1, Marker; 2, crude extract; 3, flow through; 4, first wash; 5, second wash; 6, eluate. Marker band sizes are indicated on the left in kDa. (**B**) Immunoblot of plant extracts. Crude leave extracts were separated in a 12% SDS-PAGE, transferred to a PVDF membrane and probed with Omp19 antisera. 1: WT; 2,3,4: Crude protein extracts of leaf material corresponding to 50 µg, 25 µg, and 12.5 µg of total protein, respectively. 5: 1 µg purified Omp19.

The production of plant-derived vaccines is, in principle, almost limitless and may require little or no downstream processing [30], [31]. An edible vaccine could be useful, for instance, for administration to cattle. As lipidation is not a common posttranslational modification in plants, we first studied whether a plant-made vaccine based on unlipidated (U)-Omp19 expression would be effective against brucellosis. For this purpose, we decided to use in an initial instance a fast transient expression system to obtain sufficient quantities for initial studies. Therefore, the gene encoding unlipidated U-Omp19 was cloned into the magnifection system [32] and expressed in *Nicotiana benthamiana* (tobacco) plants (**Figure 1**).

**Figure 2:**
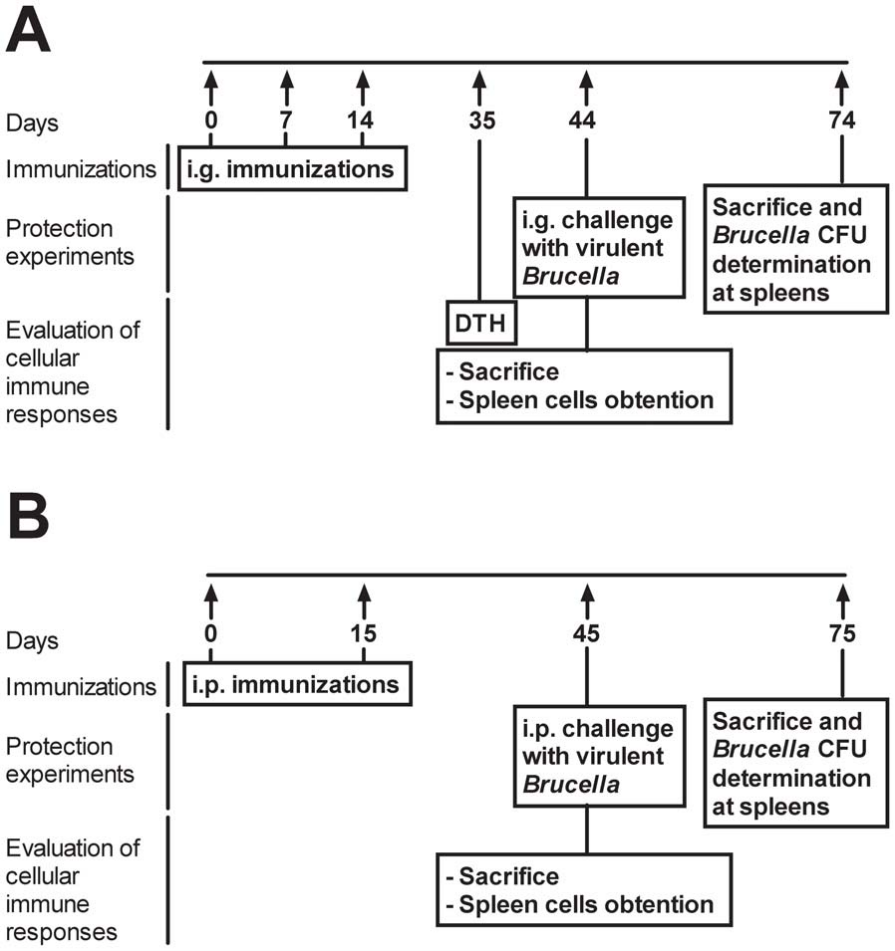
Immunization and experimental design scheme. (**A**) In order to analyze the immune responses and the elicited protective responses induced after oral immunization, mice were immunized i.g. on days 0, 7 and 14. For the protection experiments mice were challenged one month after the last immunization with virulent bacteria and 30 days later were sacrificed to analyze the bacterial burden in the spleens. For the analysis of the DTH response immunized mice were challenged with U-Omp19 3 weeks after the last immunization. Cytokine production by splenocytes was determined one month after the last immunization. (**B**) In order to analyze the immune responses and the elicited protective responses after parenteral immunization, mice were immunized i.p. on days 0 and 15. For the protection experiments mice were challenged one month after the last immunization with virulent bacteria and 30 days later were sacrificed to analyze the bacterial burden in the spleens. For the analysis of the cytokine production the spleens of immunized mice were obtained one month after the last immunization.

Firstly, we decided to evaluate the protective efficacy of the tobacco-made U-Omp19. To this aim, mice were immunized with 3 intragastric (i.g.) weekly doses of purified plant-made U-Omp19 (75 µg/dose) or with an equal amount of the purified recombinant *E. coli-*made U-Omp19. As positive and negative controls, other groups of mice were orally immunized with the attenuated vaccine strain *B. abortus* RB51 or with PBS, respectively. We used *B. abortus* RB51 as positive vaccine control since, until now, this has been the only strain used by the oral route as a positive vaccine control when animals were challenged with *B. abortus* 2308 [33]. Therefore, for comparison purposes with other published oral *Brucella* vaccine candidates, we decided to conduct the oral protection experiments using these particular strains as positive control and challenge strains respectively. One month after the last immunization all mice were challenged orally with virulent *B. abortus* 2308 and 30 days later they were sacrificed to evaluate the protection by counting the colony forming units (CFU) in their spleens (**Figure 2**). The elicited protective response was evaluated as the reduction in the splenic CFU (expressed in logarithms) determined in vaccinated mice compared with those determined in PBS-immunized mice. The purified plant-derived U-Omp19 elicited a significant protective immune response (1.26 units of protection, *P*<0.01 *vs.* PBS) when orally given to mice without adjuvant (**Table 1**). The protection level elicited by the plant-derived U-Omp19 did not differ significantly from that elicited by the recombinant *E. coli-*made U-Omp19 (1.26 units of protection *vs*. 1.22 units of protection, *P*>0.05) (**Table 1**). These results indicate that U-Omp19 is able to induce a protective immune response when orally administered without adjuvants and also that the purified recombinant plant-made U-Omp19 is as immunogenic as the recombinant *E. coli*-made U-Omp19. As this experiment was conducted without the addition of any adjuvant, it demonstrates that U-Omp19 is a self-sufficient antigen (Ag) when synthesized in a plant system as well as when synthesized in *E. coli*. Additionally, the protection levels evoked by U-Omp19 immunization did not differ significantly from the protection levels elicited by the control vaccine strain *B. abortus* RB51 (1.76 units of protection, *P*>0.05 *vs*. U-Omp19) (**Table 1**), suggesting that when developing a plant-made vaccine against brucellosis this antigen should be selected.

**Table 1:** Protection against oral *B. abortus* challenge in BALB/c mice immunized i.g. with *E. coli*- or *N. benthamiana*- derived purified U-Omp19 without adjuvants.

Vaccine (*n* = 5)	Source	Log_10_ a CFU of *B. abortus* 2308 at spleen (mean ± SD)	Units of protection
U-Omp19	*N. benthamiana*	4.30±0.52 b	1.26
U-Omp19	*E. coli*	4.34±0.12 b	1.22
*B. abortus* RB51	---	3.80±0.40 b	1.76
PBS	---	5.56±0.07 c	0

a The content of bacteria in spleens is represented as the mean log_10_ CFU ± SD per group.

b Significantly different from PBS-immunized mice *P*<0.01 estimated by Dunnett's test.

c Significantly different from *B. abortus* RB51 immunized mice *P*<0.01 estimated by Dunnett's test.

In order to evaluate if U-Omp19 would be also protective when administered in the context of crude plant material mice were immunized i.g. with freeze dried crude leaf material of transgenic tobacco leaves expressing U-Omp19. The U-Omp19 content on the crude leaf material was quantified by competitive ELISA resulting in 7.5 µg of U-Omp19 per mg of crude leaf material **(data not shown)**. A group of mice was immunized orally with U-Omp19 expressing crude leaf material (containing 75 µg of U-Omp19) while another group of mice received crude leaf material of wild type (wt) tobacco plants (that did not express U-Omp19). Other group of mice was immunized with the same amount of purified recombinant *E. coli*-made U-Omp19 as positive control while another group was immunized with PBS alone as negative control. The oral administration of crude leaf material from tobacco plants expressing U-Omp19 was able to induce significant levels of protection (1.45 units of protection, *P*<0.01 *vs.* PBS) (**Table 2**). The protection level obtained was specific to the U-Omp19 since crude leaf material from wt plants was not able to induce any protection against the challenge (−0.07 units of protection, *P*>0.05 *vs.* PBS) (**Table 2**). Moreover, when U-Omp19 was administered in the context of crude leaf material the elicited protection level was comparable to that elicited by the recombinant purified protein made in *E. coli* (1.45 units of protection *vs.* 1.15 units of protection, respectively, *P*>0.05) (**Table 2**). This result indicates that the plant material does not modify the immunogenicity of U-Omp19. The fact that U-Omp19 is a self-sufficient Ag when synthesized in a plant system provides a significant progress towards the development of an edible vaccine which will not require the addition of any adjuvants for its administration.

**Table 2:** Oral administration of U-Omp19 expressing crude leaf material without adjuvant induces protection against oral *B. abortus* challenge in BALB/c mice.

Vaccine (*n* = 5)	Log_10_ a CFU of *B. abortus* 2308 at spleen (mean ± SD)	Units of protection
*N. benthamiana*– U-Omp19	4.07±0.36 b	1.45
*N. benthamiana –wt*	5.59±0.04 c	−0.07
U-Omp19 purified from *E. coli*	4.37±0.21 b	1.15
PBS	5.52±0.09 c	0

a The content of bacteria in spleens is represented as the mean log_10_ CFU ± SD per group.

b Significantly different from PBS-immunized mice *P*<0.01 estimated by Dunnett's test.

c Significantly different from U-Omp19 immunized mice *P*<0.01 estimated by Dunnett's test.

### Oral administration of Omp19 elicits a protective response against a *Brucella* challenge without the need of external adjuvants or its lipid moiety

Bacterial lipoproteins are characterized by the presence of a lipid moiety with immunostimulatory properties at their amino-terminal end. This modification has been shown in many cases to confer them the ability to elicit specific immune responses [34]. As Omp19 has protective epitopes [16] and it is a bacterial lipoprotein [26], we hypothesized that if present the lipid moiety would increase the protective immune response in an adjuvant-free formulation. For this reason, two versions of the *E. coli*-made protein were used: the complete lipoprotein (L-Omp19) -which includes its N-terminal triacylated cysteine- and the unlipidated version (U-Omp19) obtained by cloning the protein lacking the consensus sequence for bacterial lipidation [35]. BALB/c mice were immunized with either L-Omp19 or U-Omp19 by the i.g. route without the addition of external adjuvants. As controls others groups of mice were immunized with PBS alone (negative control), U-Omp19 with CT or with the vaccine strain *B. abortus* RB51 (positive controls). Immunization with L-Omp19 without the addition of external adjuvants elicited a significant protective response against an i.g. *B. abortus* 2308 challenge (1,03 units of protection, *P*<0.05 *vs.* PBS) (**Table 3**). Yet, the protection levels elicited by L-Omp19 were roughly similar to those elicited by U-Omp19 (1.03 units of protection and 1.04 units of protection, respectively; *P*>0.05), indicating that the lipid moiety does not improve the protective response elicited by U-Omp19 when administered i.g. -without adjuvants- to mice. Furthermore, the addition of the CT adjuvant did not significantly improve the protective immune response elicited by U-Omp19 (**Table 3**). As expected the vaccine strain *B. abortus* RB51 elicited a significant protective response (1.48 units of protection, *P*<0.01 *vs.* PBS). As obtained with plant- or *E. coli*-made U-Omp19, the protection levels afforded by i.g. immunization with *E. coli*-made L-Omp19 or U-Omp19 were similar to those elicited by the attenuated vaccine strain *B*. *abortus* RB51 (*P*>0.05) (**Table 3**).

**Table 3:** Protection against oral *B. abortus* infection in BALB/c mice vaccinated orally with L-Omp19 or U-Omp19 without adjuvants or with U-Omp19+CT.

Vaccine (*n* = 5)	Adjuvant	Log_10_ a CFU of *B. abortus* 2308 at spleen (mean ± SD)	Units of protection
L-Omp19	None	4.36±0.07 c	1.03
U-Omp19	CT	4.13±0.31 b	1.26
U-Omp19	None	4.35±0.19 b	1.04
*B. abortus* RB51	None	3.91±0.64 b	1.48
PBS	None	5.39±0.16 d	0

a The content of bacteria in spleens is represented as the mean log_10_ CFU ± SD per group.

b Significantly different from PBS-immunized mice *P*<0.01 estimated by Dunnett's test.

c Significantly different from PBS-immunized mice *P*<0.05 estimated by Dunnett's test.

d Significantly different from *B. abortus* RB51 immunized mice *P*<0.01 estimated by Dunnett's test.

### Parenterally-administered U-Omp19 elicits protection against a systemic *Brucella* challenge

In order to test other routes of immunization, we decided to evaluate the protective and immune responses elicited by Omp19 when parenterally administered as an adjuvant-free formulation. BALB/c mice were intraperitoneally (i.p.) immunized with either *E. coli-*made U-Omp19 or *E. coli*-made L-Omp19 without adjuvants. As negative control other group of mice was immunized with PBS alone whereas as positive controls other groups of mice received incomplete Freund's adjuvant (IFA)-emulsified *E. coli*-made U-Omp19 or the vaccine strain *B. abortus* S19. Afterward, mice were challenged i.p. with *B. abortus* 544 strain and the elicited protective response was evaluated. Omp19 was able to elicit a significant protective immune response when given parenterally without adjuvants (*P*<0.01 *vs*. PBS) (**Table 4**). This response was independent of the protein lipidation, since there were no significant differences between the protective response elicited by U-Omp19 and L-Omp19 (1.84 or 1.56 units of protection respectively, *P*>0.05) (**Table 4**). Moreover, the protective response elicited by U-Omp19 immunization did not differ from that induced by the vaccine strain *B. abortus* S19 (1.84 *vs*. 2.27 units of protection, *P*>0.05) and the administration of the protein emulsified with IFA did not improve the elicited protective immune response (1.80 units of protection, *P*>0.05 *vs*. unadjuvanted U-Omp19) (**Table 4**).

**Table 4:** Parenteral L-Omp19 or U-Omp19 inoculation without adjuvant induce protection against *B. abortus* infection in BALB/c mice.

Vaccine (*n* = 5)	Adjuvant	Log_10_ a CFU of *B. abortus* 544 at spleen (mean ± SD)	Units of protection
L-Omp19	None	4.72±0.62 b, d	1.56
U-Omp19	None	4.44±0.26 b	1.84
U-Omp19	IFA	4.48±0.13 b	1.80
*B. abortus* S19	None	4.01±0.27 b	2.27
PBS	None	6.28±0.13 c	0

a The content of bacteria in spleens is represented as the mean log_10_ CFU ± SD per group.

b Significantly different from PBS-immunized mice *P*<0.01 estimated by Dunnett's test.

c Significantly different from *B. abortus* S19 immunized mice *P*<0.01 estimated by Dunnett's test.

d Significantly different from *B. abortus* S19 immunized mice *P*<0.05 estimated by Dunnett's test.

The fact that *E. coli*-made U-Omp19 induced similar levels of protection to those elicited by the plant-derived U-Omp19 ensures that the observed self-adjuvanticity is not a result of an *E. coli-*derived contaminant. To confirm this fact, the protective capacity of U-Omp19 was evaluated in Toll like receptor 4 knock out (TLR4^−/−^) mice. TLR4^−/−^ or wt C56BL/6 mice were immunized with *E. coli*-made U-Omp19 or PBS and the elicited protective response was evaluated. U-Omp19 elicited a significant protective response (*P*<0.01 *vs.* PBS) either in wt or TLR4-deficient mice (**Table 5**), indicating that this receptor is not involved in the protective response induced by U-Omp19 and that the protein is protective in a more resistant mouse strain: C57BL/6 [36].

**Table 5:** Protection against *B. abortus* in C57BL/6 wt or TLR4^−/−^ mice immunized with U-Omp19 without adjuvants.

Strain (*n* = 5):		wt		TLR4^−/−^	
Vaccine	Adjuvant	Log_10_ a CFU of *B. abortus* 2308 at spleen (mean ± SD)	Units of protection	Log_10_ a CFU of *B. abortus* 2308 at spleen (mean ± SD)	Units of protection
U-Omp19	None	4.60±0.34 b	1.12	4.23±0.20 c	1.03
PBS	None	5.72±0.06	0	5.26±0.54	0

a The content of bacteria in spleens is represented as the mean log_10_ CFU ± SD per group.

b Significantly different from wt PBS-immunized mice *P*<0.01 estimated by Student’s test.

c Significantly different from TLR4^−/−^ PBS-immunized mice *P*<0.01 estimated by Student’s test.

### The lipid moiety of Omp19 improves the elicited humoral immune response after oral or systemic immunization

**Figure 3:**
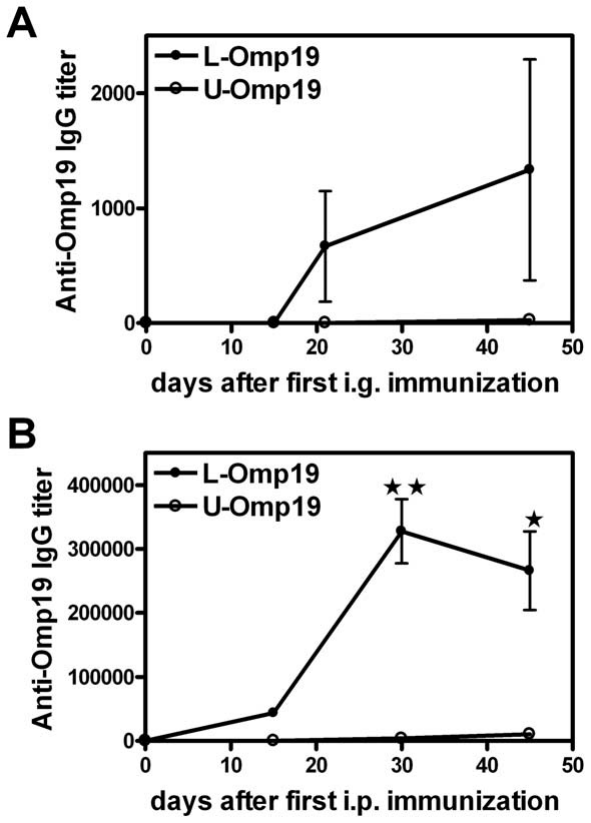
Kinetic of the specific humoral responses elicited after administration of adjuvant-free Omp19. BALB/c mice were immunized i.g. (**A**) or i.p. (**B**) with L-Omp19 (•) or U-Omp19 (**○**) without adjuvants as indicated in materials and methods and the kinetic of the Omp19 specific humoral response elicited after immunizations was determined. Serum samples were obtained at the indicated time after the first immunization. Omp19-specific IgG Ab titers were determined by ELISA. Each point represents the mean ± S.E.M of the Ab titer from 5 mice per group. (★) and (★ ★) significantly different from the U-Omp19 immunized group, (*P*<0.05) and (*P*<0.01), respectively. This experiment was conducted three times with similar results.

To further characterize the immune response elicited by the administration of unadjuvanted Omp19 we first evaluated the elicited specific humoral response in immunized mice. The kinetics of specific serum immunoglobulin (Ig) G antibodies production in immunized mice was determined. Immunization with *E. coli*-made L-Omp19 induced a mild specific humoral response when administered i.g., with maximal peak of titers arising 45 days after the first immunization, whereas *E. coli*-made U-Omp19 induced almost undetectable titers of specific IgG (**Figure 3A**), while no specific anti-Omp19 IgA was detected in feces from any orally immunized mice (data not shown). When the humoral immune responses elicited by i.p. immunization with *E. coli*-made U-Omp19- or L-Omp19 were evaluated, a similar result to orally immunized mice was obtained. The protein portion of Omp19 elicited a slightly undetectable specific humoral immune response whereas the lipidated protein elicited elevated anti-Omp19 IgG titers (**Figure 3B**). This result indicates that the lipid moiety could improve the humoral immune response against Omp19 but this improvement does not have any effect on the protection levels (**Tables 3** **and** **4**). These results are in agreement to the already described poor contribution of the humoral immune response in the protective responses against smooth strains of *Brucella* spp. [37].

### The protein portion of Omp19 elicits a specific Th1-Th17 immune response when orally delivered without adjuvant and a specific Th1 immune response when administered by the systemic route

**Figure 4:**
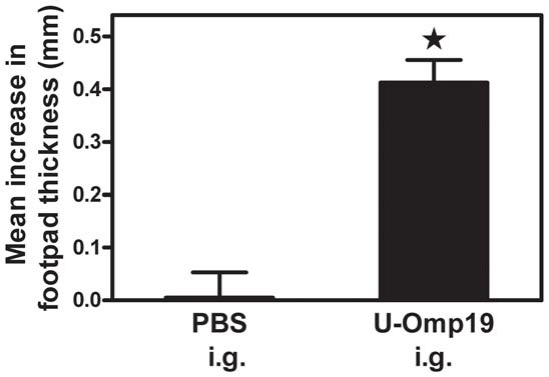
Specific DTH response elicited after i.g. administration of adjuvant-free Omp19. Three weeks after the last i.g. immunization mice were intradermally challenged with U-Omp19 in their left footpad and an equal volume of saline into their right footpad. DTH response was measured at 72 h following injection of antigen. Each bar represents the mean increase in the footpad thickness ± SEM from 5 mice per group. (★) Significantly different from the mean increase thickness measured in PBS immunized mice (*P*<0.05).These experiments were conducted three times with similar results.

**Figure 5:**
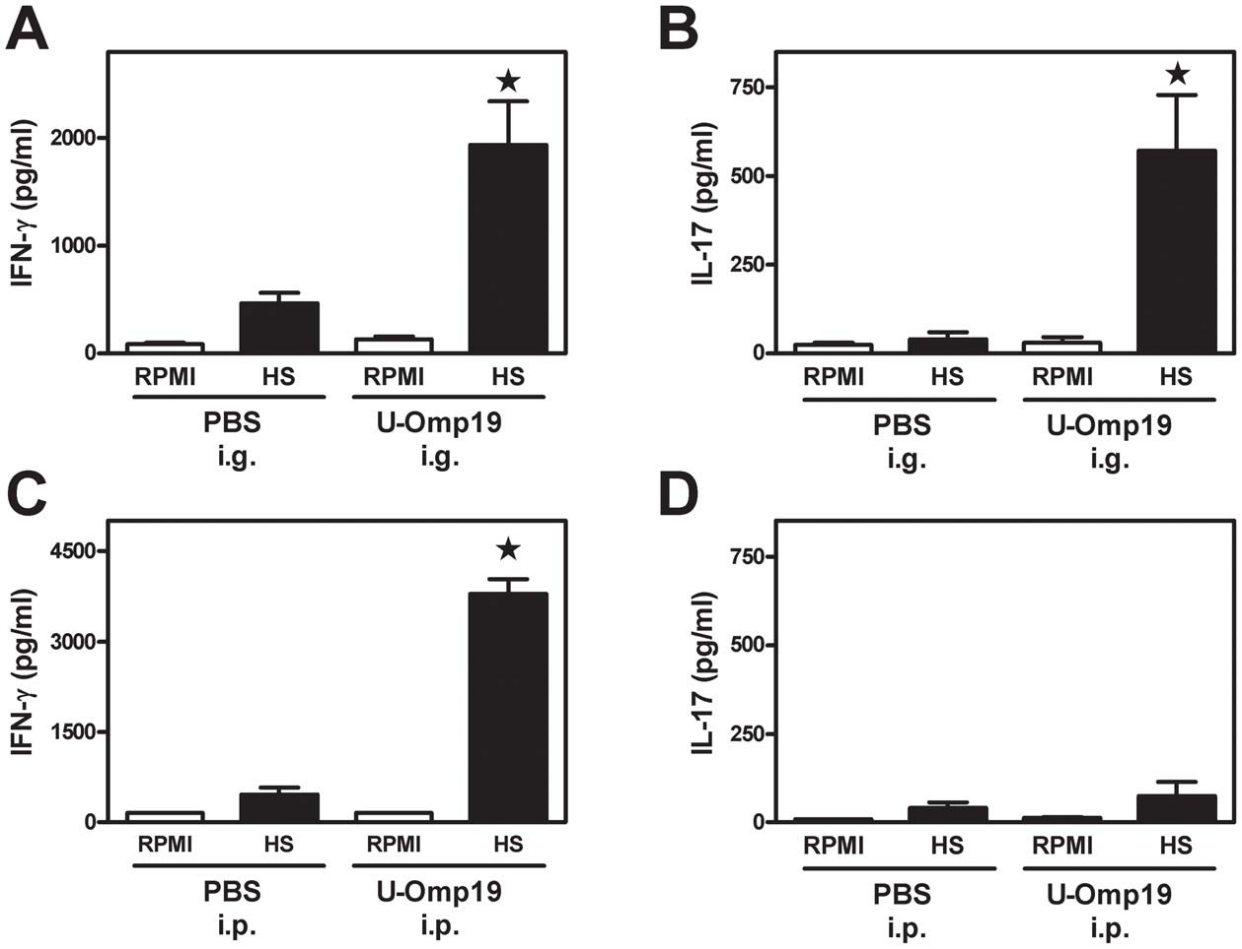
Cellular immune responses elicited after i.g. or i.p. administration of adjuvant-free Omp19. Mice were immunized with U-Omp19 or PBS i.g. (**A–B**) or i.p. (**C–D**) as indicated in materials and methods and in Figure 1. Specific production of IFN-γ (**A and C**) and IL-17 (**B and D**) by splenocytes of i.g. or i.p. U-Omp19 immunized mice was evaluated 30 days after the last immunization. Spleen cells (4×10^6^ cells/ml) were cultured with HS (20 µg/ml) or complete medium alone (RPMI) for 72 h. Each sample was assayed in duplicate wells. Cytokines in culture supernatants were measured by sandwich ELISA. Data represent the mean ± SEM from each group of five mice. (★) Significantly different from the cytokine level induced by the same stimulus in PBS immunized mice (*P*<0.05). These experiments were conducted three times with similar results.

In order to characterize the cellular immune response induced *in vivo* after oral U-Omp19 immunization, the delayed type hypersensitivity (DTH) response was determined in immunized mice. After an intradermal U-Omp19-challenge a significant DTH response (*P*<0.05 *vs*. PBS-immunized mice) was observed in i.g. *E. coli*-made U-Omp19-immunized mice (**Figure 4**). To further characterize the elicited cellular immune response we tried to emulate the *in vivo* situation where T cells are confronted with the whole *Brucella*, therefore we decided to use for the recall experiments a hot saline extract of *Brucella* (HS) that contains the bacterial proteins including native Omp19 [26]. Spleen cells from immunized mice were cultured *ex vivo* with HS or with complete culture medium alone (RPMI) as control. When splenocytes from i.g. U-Omp19-immunized mice were stimulated *in vitro* with HS they produced significant amounts of the cytokines IFN-γ and IL-17 (*P*<0.05 *vs.* PBS-immunized mice in response to the same stimulus, **Figure 5A and B**, respectively). On the contrary, the same splenocytes did not produce IL-2, IL-4 or IL-10 after HS *in vitro* stimulation (data not shown).

In the case of systemic immunizations, splenocytes from i.p. *E. coli*-made U-Omp19-immunized mice secreted significantly higher amounts of IFN-γ in response to HS than splenocytes from PBS-immunized mice in response to the same stimulus (*P*<0.05) (**Figure 5C**). However, HS *in vitro* stimulation did not stimulate the production of IL-2, IL-4, IL-10 or IL-17 by the splenocytes of either i.p. U-Omp19- or PBS-immunized mice (data not shown and **Figure 5D**). All together these results indicate that U-Omp19 has the capacity to elicit a significant cellular mixed Th1-Th17 immune response without the need of adjuvant's help when administered by the oral route and a specific Th1 immune response when administered i.p. to mice.

### U-Omp19 activates dendritic cells in vivo

**Figure 6:**
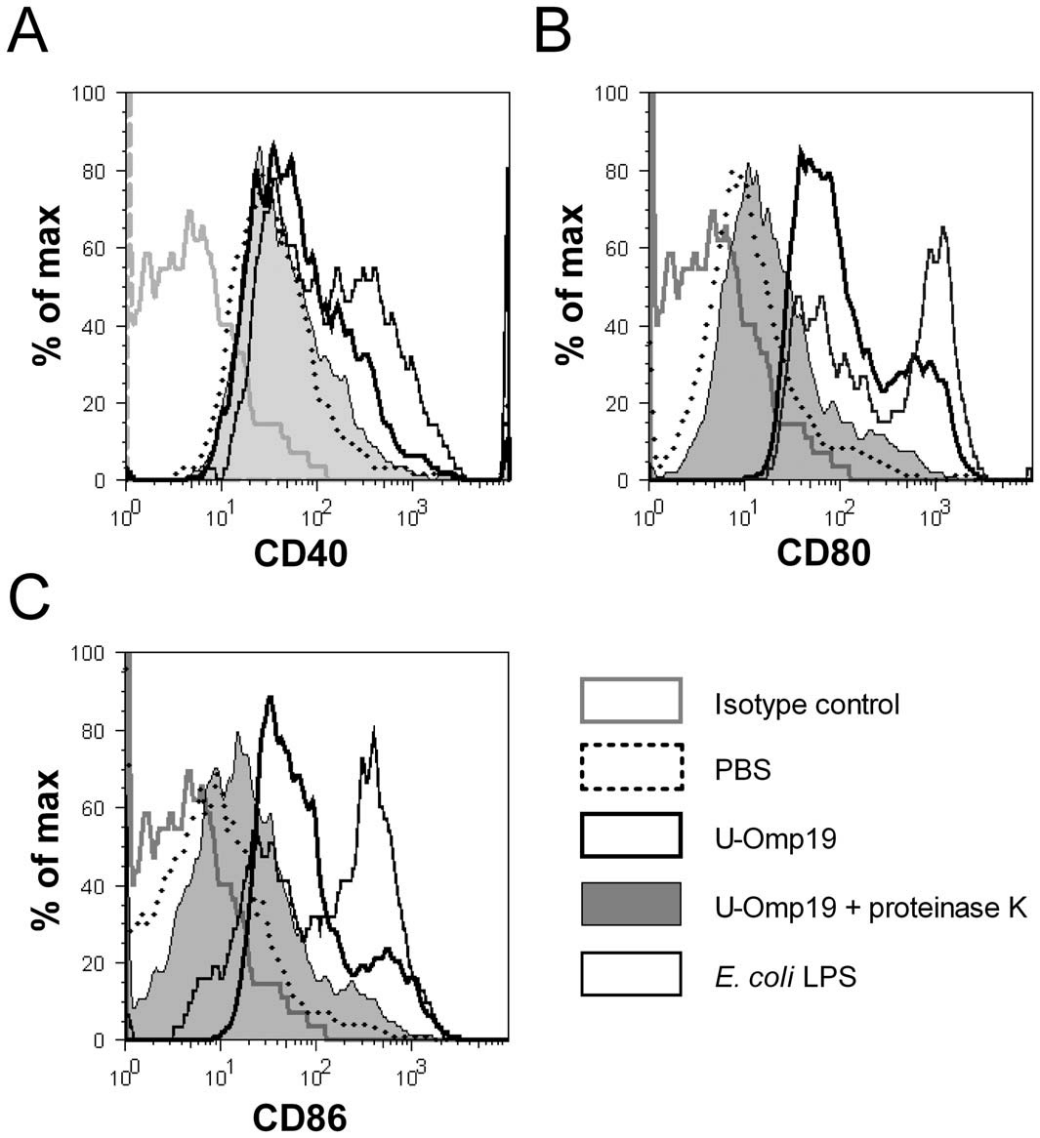
*In vivo* activation of DCs after U-Omp19 injection. U-Omp19 either untreated or digested with proteinase K, *E. coli* LPS or PBS alone was injected i.v. to BALB/c mice. Splenic CD11c^+^ DCs were analyzed for their activation status 20 h after injection by assessing the surface expression of (**A**) CD40, (**B**) CD80 and (**C**) CD86 molecules by flow cytometry. This experiment was conducted three times with similar results. Histograms display results from one representative experiment.

The initiation of an immune response mainly involves the capture and processing of an antigen by dendritic cells (DC) that are activated by local stimuli. After activation they migrate to lymphoid organs and up-regulate the expression of various co-stimulatory molecules improving the antigen specific adaptive immune response [38]. The addition of an adjuvant into a subunit vaccine formulation has usually the purpose of activating DC at the site of inoculation and thereby inducing a strong and effective specific immune response against the co-administered antigen [39]. The fact that U-Omp19 did not require the addition of adjuvants for the induction of effective protective responses prompted us to analyze if the administration of U-Omp19 has an effect on the maturation status of DC *in vivo*. BALB/c mice were intravenously (i.v.) injected with *E. coli*-made U-Omp19 and 20 h later the maturation status of their splenic DC was assessed by flow cytometry. As controls other groups of mice received by the same route: *i*) proteinase K-digested U-Omp19 control, *ii*) PBS alone (basal control) or *iii*) *E. coli* LPS (full-maturation control). The maturation status of splenic DC was evaluated analyzing the surface expression of co-stimulatory molecules on CD11c^+^ cells. After U-Omp19 administration the expression of CD40, CD80 and CD86 molecules was up-regulated on splenic DC when compared to their basal expression levels on splenic DC from PBS-injected-mice (**Figure 6A, B and C**, respectively). Conversely, after being fully-digested with proteinase K, the U-Omp19 ability to stimulate DC *in vivo* was completely abrogated. On the contrary, the same protein digestion treatment did not affect the activity of *E. coli* LPS which, as expected, up-regulated the expression of the studied co-stimulatory molecules (**Figure 6** and **data not shown**). These results indicate that U-Omp19 is endowed with *in vivo* DC-stimulating activity. This DC-stimulatory activity might explain, at least in part, the lack of need of adjuvants to induce a U-Omp19-specific and effective immune response when the protein moiety is given to mice alone.

### IL-17 plays a key role in the protective immune response elicited by the unadjuvanted oral administration of U-Omp19

The role of CD4^+^ T cells and the cytokine IFN-γ in protective responses against *Brucella* has been well established. Nevertheless, the impact of IL-17 on *Brucella* infection, as well as its role on vaccine-induced protective responses has never been evaluated. Having in mind that animals immunized by the oral route with U-Omp19 induced a Th17 immune response *in vitro* we decided to investigate the role of IL-17 and CD4^+^ T cells in the elicited protective response against *Brucella* after oral immunizations with U-Omp19. Different groups of mice were orally immunized with *E. coli*-made U-Omp19 or PBS and received different treatments before or after the oral challenge with *B. abortus* 2308. In order to abolish the CD4^+^ T cells primed by the immunizations, one group of U-Omp19 immunized mice was treated with anti-CD4 monoclonal antibody (mAb) prior to the challenge. This treatment eliminated all the mature CD4^+^ T cells at the time of challenge. Other groups of mice were treated with anti-IL-17 mAb immediately after the challenge and every 6 days until their sacrifice. One month after the oral *Brucella* challenge all mice were sacrificed and the elicited protective response was evaluated. The ability of U-Omp19 to elicit a protective response when orally administered was lost when the mature CD4^+^ T cell population was depleted *in vivo* prior to the challenge (0.32 units of protection, *P*>0.05 *vs*. PBS) (**Table 6**). At the same time, U-Omp19 immunized-mice treated with an irrelevant isotype matched mAb exhibited a significant level of protection (1.14 units of protection, *P*<0.01 *vs*. PBS) (**Table 6**). The protection level elicited in the latter group was statistically higher than the one induced by the CD4^+^ T cell-depleted group (*P*<0.01) indicating that the CD4^+^ T cell population induced after U-Omp19 vaccination has a main role in eliciting oral immune protection (**Table 6**).

**Table 6:** Protective responses against oral *Brucella* infection elicited by U-Omp19 oral vaccination are mediated by CD4^+^ T cells and IL-17.

Vaccine (*n* = 5)	Treatment	Log_10_ a CFU of *B. abortus* 2308 at spleen (mean ± SD)	Units of protection
U-Omp19	Isotype Control	4.36±0.21 b	1.14
U-Omp19	Anti - CD4	5.18±0.41 c	0.32
U-Omp19	Anti - IL-17	5.03±0.58 d	0.47
PBS	Isotype Control	5.50±0.09 c	0
PBS	Anti - IL-17	5.66±0.30 c	−0.16

a The content of bacteria in spleens is represented as the mean log_10_ CFU ± SD per group.

b Significantly different from PBS-immunized mice *P*<0.01 estimated by Dunnett's test.

c Significantly different from U-Omp19 immunized (Isotype Control-treated) mice *P*<0.01 estimated by Dunnett's test.

d Significantly different from U-Omp19 immunized (Isotype Control-treated) mice *P*<0.05 estimated by Dunnett's test.

In the same way, neutralization of IL-17 significantly reduced the elicited protective response (0.47 units of protection, *P*>0.05 *vs*. PBS and *P*<0.05 *vs*. U-Omp19 immunized mice treated with an isotype matched irrelevant antibody) (**Table 6**). On the contrary, IL-17 neutralization during the infection did not influence at all the persistence and multiplication of this bacteria in PBS-immunized mice, since at the moment of the sacrifice the bacterial burden was not different between PBS-immunized mice treated with anti-IL-17 and an isotype matched irrelevant antibody (*P*>0.05) (**Table 6**). These findings provide *in vivo* evidence that IL-17 plays a crucial role in the adaptive immune response against an oral infection with the intracellular bacteria *B. abortus.*

## Discussion

A great deal of effort has been directed toward the replacement of existing whole cell or formalin-inactivated vaccines with subunit vaccines that may be safer and more effective than existing vaccines [40]. In addition, other efforts are directed at developing alternatives to traditional vaccine delivery, including mucosal (oral) delivery of plant-derived vaccines [31]. As *Brucella* infections mainly involve the bacterial entry through the mucosal routes, the development of successful approaches for oral vaccination could radically alter the current scene of brucellosis. Most published studies have evaluated the use of attenuated *Brucella* strains [33], [41], [42] or live vectors expressing *Brucella* antigens [43], [44] or carrying DNA plasmids for DNA vaccine delivery at the mucosal gut [45]. At present, only 3 recombinant proteins of *Brucella* have been evaluated as oral vaccine candidates: the choloylglycine hydrolase (CGH) [46], Omp16 and Omp19 [16], [47]. To our knowledge there are no reports describing the use of an edible vaccine made in plants against *Brucella.*

Plant-derived oral vaccines are a very suitable approach with many advantages, including their ability to stimulate systemic and mucosal immune responses, their simplified large-scale production and storage (eliminating frozen stocks), and their improved safety due to the lack of human pathogens or microbial toxin contamination [2]. Our results indicate that plant-expressed U-Omp19 is able to induce significant protective immune responses when administered to mice by the oral route as a purified protein as well as within the crude leaf material of transgenic tobacco plants. In both cases the achieved protection levels were equivalent to those elicited by the purified *E. coli*-made U-Omp19, indicating that the plant-made U-Omp19 has similar self-adjuvanting properties to its homologous expressed and purified from *E. coli*.

U-Omp19 is a very promising vaccine candidate, since it could be expressed in an edible plant and administered with the food to animals. In the case of bovines, it has been described that rumination could be exploited for vaccine improvement, since if the vaccine antigen is expressed by a fibrous feed like alfalfa the nasopharyngeal immune tissues have a prolonged exposure to the vaccine during the cud chewing [3]. Yet, in cattle and other ruminants protein administration by the oral route would have a negative side because of the enhanced antigen degradation in the rumen, previous to the passage to the intestine [3]. However, antigens expressed in plant would be protected by bioencapsulation, enhancing the antigen delivery to the gut-associated lymphoid tissue [30], [31], [48].

A major restrictive factor for the development of plant-derived oral vaccines is the availability of efficient and safe adjuvants that work at mucosal sites. Induction of immune responses following mucosal immunization is usually dependent on the co-administration of appropriate adjuvants that, overcoming tolerance, can initiate and support the transition from innate to adaptive immunity [49]. However, many adjuvants are associated with toxicity or side-effects and their use also increases the cost of vaccine manufacture and introduces complexity [28]. Therefore, the use of adjuvant-free vaccines would be considered a very worthwhile approach for oral and systemic vaccination. Auspiciously, U-Omp19 administered without adjuvants is able to induce protective immunity against *Brucella* in BALB/c mice when administered by the oral route as well as by the systemic route. This protective capacity is neither improved by the lipidation of the protein nor by the addition of external adjuvants (CT when orally administered or IFA when i.p. delivered). The fact that the protein moiety of Omp19 has the ability to induce the protective responses by itself is a very favorable attribute in the vaccine field, since the lack of adjuvants in the formulation would reduce adjuvant associated drawbacks such as toxicity, side effects, cost, etc., and the U-Omp19 production is beyond any doubt easier and more economical than the one of L-Omp19 [16].

U-Omp19 elicits straightly similar protection levels to those conferred by the current used attenuated vaccine strains by the oral route or by the parenteral route (*B. abortus* RB51 and *B. abortus* S19, respectively), indicating that U-Omp19 would be a rational component in a subunit vaccine formulated with only few protective antigens against brucellosis. Yet, this should be proven in the final hosts of *Brucella* spp. (cattle, goats, sheep and swine).

The ability of U-Omp19 to elicit a protective response against a *B. abortus* infection without the addition of adjuvants was not only observed when orally administered but also when i.p. administered to mice. These results indicate that this protein can act as a self-adjuvant on the mucosal as well as on the systemic immune system. Furthermore, the elicited systemic protective response was not only protective against *B. abortus* infections, but also against *B. melitensis* and *B. suis* infections (data not shown). Taking all these results into account, U-Omp19 would be a powerful component of a subunit vaccine against brucellosis, which could be useful for rapid vaccination of different *Brucella* affected livestock species and be administered by different immunizations routes without the need of external adjuvants.

As the activation of DCs plays a critical role in the initiation of immune responses, we evaluated the ability of U-Omp19 to activate this cell population. Our results indicate that U-Omp19 activates DCs *in vivo* as indicated by the up-regulation of the expression of CD40, CD80 and CD86 molecules on DC membrane. This activity is completely dependent on the presence of the intact protein, since it was lost when the complete degraded protein with proteinase K was used as stimulant.

After oral or i.p. administration of Omp19 a mild specific humoral immune response is induced in L-Omp19 immunized mice. On the contrary, in U-Omp19-immunized mice the anti-Omp19 IgG antibodies are practically undetectable in serum of immunized mice. The lack of a specific humoral response elicited by i.g. U-Omp19 administration compared to L-Omp19 immunizations by the same route together with the similarities in the protective immune responses evoked by the lipidated or unlipidated versions of the protein are in concordance with the usually associated scant participation of antibodies in the protective immune responses against smooth strains of *Brucella* spp., such as *B. abortus* [9].

Throughout all this manuscript there are many results that validate that the measured self-adjuvanting properties of U-Omp19 are not due to *E. coli* contaminants, mainly LPS, but to a feature of the protein moiety. Firstly, the protection level induced by the oral administration of tobacco derived purified U-Omp19, the crude leaf material expressing this protein or the *E. coli*-made U-Omp19 was equal. Secondly, *E. coli*-made U-Omp19 induced significant and equivalent levels of protection in wt and TLR4^−/−^ C57BL/6 mice, indicating that the TLR4 receptor is not involved in the ability of U-Omp19 to elicit a protective response. Last but not least, the abrogation of the *in vivo* DC-stimulating properties of U-Omp19 by the complete protein degradation with proteinase K in combination with the lack of effect of the same treatment on the *E. coli*-LPS activity provided evidence that the measured activity resides in the U-Omp19 protein moiety.

Because of its intracellular residence, effective immune responses against *Brucella* include predominantly cell-mediated immunity. Furthermore, Th1 and CTL responses are critical components involved in anti-Brucella protection. Principally IFN-γ, which activates macrophages for more efficient killing and inhibition of replication of intracellular microbial pathogens, is generally considered crucial in the battle against this illness [50]. The *in vivo* CD4^+^ T cell subset depletion experiment clearly showed that oral protection against *B. abortus* elicited by U-Omp19 is mainly attributed to CD4^+^ T cells. Moreover, oral administration of U-Omp19 induced a mixed Th1-Th17 specific cellular immune response, while systemic administration of U-Omp19 elicited a Th1 immune response. In contrast to Th1 differentiation, which depends on IL-12, Th17 differentiation in mice requires IL-6 and TGF-β *in vivo*. DC can determine whether nonresponsiveness (tolerance) or an active immune response occurs to a particular antigen, as well as determining whether a T helper (Th) 1, Th2, Th17 or a regulatory response predominates [51], [52]. As there are many differences in terms of cell distribution and cytokine expression between gut and spleen it would be possible that -when orally delivered - Omp19 can bind and activate a specific cell population present at the gut that is not present at the spleen that positively regulated the differentiation of interleukin 17-producing T helper cells.

Recently, the role of Th17 responses in infectious diseases has started to be elucidated. It has been shown that Th17 responses are important for the host defense against many microorganisms, although they can also contribute to immunopathology during infection [53], [54]. In infections caused by bacteria and fungi, the pathogen-induced Th17 response has been reported as an important mediator of protective mucosal host defense [53], [54]. At present, the role that IL-17 might play in the clearance of *Brucella* spp. is largely unknown. As oral administration of U-Omp19 induces a Th17 immune response *in vitro* we decided to study its *in vivo* role in the elicited protection. Unprecedented, our results demonstrate that IL-17A neutralization during the challenge period abrogates U-Omp19 vaccine efficacy, while does not affect the bacterial burden in PBS-immunized mice.

*Brucella* is an intracellular facultative pathogen that infects professional and non-professional phagocytes, including macrophages, neutrophils, placental trophoblasts, DC and epithelial cells [4]. After being internalized, *Brucella* builds its replicative niche where it resists the intracellular killing and replicates. *Brucella* intracellular fate is crucial to cause illness and involves several important virulence factors, which allow the bacterium to survive and proliferate within a membrane compartment, called the *Brucella*-containing vacuole. This vacuole interacts transiently with endosomes and fuses with the ER membrane establishing a replicative compartment and causing a chronic infection [4]. There are evidences that IL-17 plays a protective role against the primary infection of some intracellular bacteria, such as *Francisella tularensis* and *Listeria monocytogenes* [55]–[57], while it has been shown to have a limited role with other intracellular primary infections, such as tuberculosis [58] or a controversial role like in *Salmonella enterica* [59]–[61]. However, recent evidence supports a critical role of Th17 cells in vaccine-induced protection to both extracellular and intracellular infections, such as *Staphylococcus aureus*, *Candida albicans*, *Bordetella pertusis*, *Mycobacterium tuberculosis*, *Pseudomonas aeuruginosa* [23], [58], [62]–[64]. To our knowledge, this is the first demonstration that vaccine-induced IL-17 is critical against an oral delivery challenge with virulent intracellular bacteria. In the case of *Brucella* infection, our results indicate different and important new discoveries: i) IL-17 does not contribute to reduce the bacterial burden in non vaccinated young mice, at least during the first month of infection and ii) IL-17 plays a central role in vaccine mediated anti-*Brucella* immunity.

In conclusion, altogether our results demonstrate that an oral vaccine based on U-Omp19 induces protection against mucosal challenge with the intracellular bacteria *B. abortus* by inducing an adaptive IL-17 immune response.

## Materials and Methods

### Ethics Statement

All experimental protocols of this study were conducted in strict accordance with international ethical standards for animal experimentation (Helsinki Declaration and its amendments, Amsterdam Protocol of welfare and animal protection and National Institutes of Health, USA NIH, guidelines: Guide for the Care and Use of Laboratory Animals). All surgeries were performed under sodium pentobarbital anesthesia. They were performed by the premise of minimizing the suffering to which animals are exposed and use the minimum number of experimental animals to ensure statistically significant results. The protocols of this study were approved by our Institutional Committee for the Care and Use of Laboratory Animals (CICUAL) from the University of Buenos Aires (Permit Number: 1100).

### Mice

All mice experiments were performed using 8–10 week-old-female specific-pathogen-free mice. Mouse strains were: BALB/c mice (obtained from Universidad Nacional de La Plata, Argentina), C57BL/6 wt mice (provided by the Federal University of Minas Gerais, UFMG; Belo Horizonte, Brazil) and genetically deficient TLR4^−/−^ C57BL/6 mice (provided by S. Akira, Osaka University, Japan). Mice were housed in appropriate conventional animal care facilities and handled following international guidelines required for animal experiments.

### Bacterial strains

*B. abortus* strain 2308 and *B. abortus* strain 544 were used in the challenge experiments. *B. ovis* strain REO 198 was used for production of the antigenic preparation Hot Saline (HS) and *B. abortus* RB51 and *B. abortus* S19 were used as vaccine controls. All strains were obtained from our own laboratory collection [13], [15], [65] derived from the *Brucella* culture collection (INRA-Nouzilly, France). Bacterial growth and inocula preparation were performed as previously described [65], [66]. *E. coli* BL21(DE3) (Novagen, Madison, WI) was used for recombinant protein expression.

### Antigen production

The recombinant lipidated (L-) and unlipidated (U-) Omp19 proteins were obtained as previously described [35]. Briefly, using *B. abortus* 544 genomic DNA as template, the *B. abortus* Omp19 gene sequences were cloned into the pET 22b^+^ vector (Novagen, Madison, WI). The unlipidated version was cloned using different forward primers, resulting in the gene sequence lacking the putative signal peptide and the N-terminal cysteine. After cloning, the resulting plasmids (pET-L-Omp19, pET-U-Omp19), contained the genes with a COOH-terminal 6× histidine tag. The expression of recombinant Omps in *E. coli* BL21(DE3) was induced with isopropyl β-D-thiogalactoside (1 mM). The lipidated protein, L-Omp19, was isolated from bacterial membranes whereas the unlipidated protein, U-Omp19, was isolated from bacterial cytoplasm. Purification was performed by affinity chromatography with a Ni-NTA resin (Qiagen, Dorking, U.K.). The process of expression and purification was monitored by SDS-PAGE followed by silver staining. The identity of the Omps was confirmed by western blot developed with anti-Omp19 specific mAbs. In some experiments, a U-Omp19 enzymatically digested preparation was used as control. U-Omp19 was treated with proteinase K-agarose from *Tricirachium album* (Sigma) for 2 h at 37°C following manufacturer's indications. The enzyme immobilized in agarose was then centrifuged out (2000 g, 5 min) and the supernatants were incubated for 1 h at 60°C to inactivate any fraction of soluble enzyme. The complete digestion of the proteins was checked by SDS-PAGE, followed by coomassie blue staining.

In the *in vitro* cell stimulation assays a hot saline extract (HS) from *B. ovis* REO 198, that is enriched in *Brucella* Omps and that contains the native Omp19 [26], was utilized as stimulus. HS was obtained as previously described [66].

Protein concentration was determined by the bicinchoninic acid assay with bovine serum albumin as a standard (Pierce, Rockford, IL). LPS contamination was eliminated with Sepharose-polymyxin B. Endotoxin determination was performed with *Limulus* amoebocyte assay (LAL) (Associates of Cape Cod, Woods Hole, MA). All protein preparation contained <0.25 endotoxin U/µg protein.

### Omp19 expression in *N. benthamina* plants

For the transient plant expression the coding sequence of *U- omp19* was PCR amplified using primers PO19-102 (5′-TTTGGTCTCAAGGTATG CAGAGCTCCCGGCTTGG-3) and PO16-201(5′-GCTCTAGATCAGTGGTGGTGGTGTGGTGCTC-3′). The construct was cloned into pCRblunt (Invitrogen, Karlsruhe, Germany) and sequenced for integrity check. Subsequent cloning of the *Xba*I*/Bsa*I- fragment into vector pICH10990 resulted in vector termed pO19-6122. The full-length coding sequence of Omp19 was cloned by pasting a *Nco*I/*Xba*I fragment from vector pO19-3211 into the 3′-module pICH11533. Transformation, infiltration, and purification were carried out according to Pasquevich *et al*. 2010 [47].

### Immunizations and experimental design

#### Intragastric (i.g.) immunization of mice

Groups of 5 BALB/c mice were immunized on days 0, 7 and 14 i.g. as previously described [16], [46]. Different groups received the following treatments: i) 75 µg of purified recombinant U-Omp19 in 200 µl of bicarbonate buffer (HCO_3_Na 0.1M pH 8), ii) 75 µg of purified recombinant *N. benthamiana* made U-Omp19 in 200 µl of bicarbonate buffer, iii) 75 µg of purified recombinant *E. coli* U-Omp19 mixed with 5 µg of cholera toxin (CT) (Sigma-Aldrich) in 200 µl of bicarbonate buffer as positive control, iv) 75 µg of L-Omp19 in 200 µl of bicarbonate buffer or v) 200 µl PBS plus bicarbonate buffer as negative-control. For the protection experiments an extra group of mice received iv) a single dose i.g. of 0.5×10^9^ CFU of live *B. abortus* RB51 (positive control).

In some protection experiments mice were immunized i.g. with transgenic crude leaf material of *N. benthamiana* plants. In those experiments mice received 3 i.g. doses of i) 10 mg of freeze-dried transgenic *N. benthamiana* leaf powder (containing ≅75.8 µg of U-Omp19) suspended in bicarbonate buffer, ii) 10 mg of freeze-dried non transgenic *N. benthamiana* (*wt*) leaf powder suspended in bicarbonate buffer, iii) 75.8 µg of purified *E. coli*-made recombinant U-Omp19 in bicarbonate buffer or iv) PBS plus bicarbonate buffer.

#### Parenteral immunization of mice

Groups of 5 BALB/c or C57BL/6 mice were immunized on days 0 and 15 intraperitoneally (i.p.) with 30 µg of the specified antigen. As control some mice were immunized i.p. with U-Omp19 emulsified in IFA. In addition other groups of mice were immunized with PBS as negative control and for the protection experiments as positive control an extra group of mice received i.p. a single dose of the vaccine strain *B. abortus* strain 19 (1×10^4^ CFU/dose).

Sera samples were obtained from blood samples collected from the retro-orbital plexus and fecal pellets from each mouse were collected on days 0, 15, 30 and 45 after the first immunization. After immunizations, mice were used to evaluate the elicited protective immune response or the specific cellular immune responses (cytokines production or DTH response).

### Antibody detection

Anti-Omp19 IgG, IgG1 and IgG2a specific antibody titers in the sera of immunized mice were measured with a specific indirect ELISA, as previously described [16]. Anti-Omp19 IgA was analyzed in Fecal extracts by indirect ELISA using a goat anti-mouse IgA-specific horseradish peroxidase conjugate (Santa Cruz Biotechnology, Santa Cruz, CA). Fecal extracts were prepared by suspending 5 fecal pellets in 0.5 ml of extraction buffer (100 µg/ml soybean trypsin inhibitor (Sigma), 10 mg/ml bovine serum albumin (SIGMA) and 30 mM disodium EDTA in PBS, pH = 7.6). After homogenization and centrifugation at 4°C, the supernatants of the fecal extracts were used for IgA determination in feces.

### Protection experiments

One month after the last immunization mice were challenged with virulent *Brucella* by the same route of the immunizations. Intragastrically (i.g.) immunized mice were challenged i.g. with 3×10^8^ CFU of *B. abortus* 2308 while i.p. immunized mice were challenged with 4×10^4^ CFU of *B. abortus* 544 or *B. abortus* 2308. One month after challenge spleens where aseptically removed, homogenized in sterile PBS, diluted, plated and incubated as described [65] to determine the number of *Brucella* colonies. Results were represented as the mean log CFU ± SD per group. Units of protection were calculated subtracting the mean log_10_ numbers of CFU in the experimental group from the mean log_10_ numbers of CFU in the PBS-immunized group. Each experiment was repeated three times.

### Cellular immune responses

#### DTH Response

DTH tests were performed as an *in vivo* index of the elicited cell mediated immunity. Three weeks after the last i.g. immunization mice received intradermally 30 µg of U-Omp19 into the left footpad while an equal volume of vehicle (saline) was injected into their right footpad. After 72h the DTH reaction was quantified by measuring the difference between the footpad thicknesses using a digital caliper with a precision of 0.01 mm. The mean increase in footpad thickness (mm) was calculated according to the following formula: left footpad thicknesses (U-Omp19) − right footpad thicknesses (saline).

#### Cytokine production

Spleen cells from immunized mice were obtained 1 month after the last immunization and were stimulated as previously described [16]. Briefly, single cell suspensions of spleen cells from immunized and control mice were cultured in duplicate at 4×10^6^ cells/ml in RPMI 1640 (Gibco BRL, Life Technologies, Grand Island, N.Y.) supplemented with 10% fetal calf serum (Invitrogen Life Technologies), 1 mM sodium pyruvate, 2 mM L-glutamine, 100 U of penicillin/ml, 100 µg of streptomycin/ml (complete medium) with stimuli. The different stimuli were: HS (20 µg/ml) or complete medium alone. After 72 h of incubation at 37°C in a humidified atmosphere (5% CO_2_ and 95% air) cell culture supernatants were collected and immediately stored at −80°C until further analysis. Cytokine production was analyzed using mouse ELISA kits according to the manufacturer's instructions: IFN-γ, IL-2, IL-4, IL-5, IL-10 (Pharmingen, San Diego, CA, USA) and IL-17 (R&D Systems, Minneapolis, MN, USA).

### *In vivo* analysis of DC maturation

*In vivo* activation of DCs was evaluated with flow cytometry by measuring the expression of various surface markers. BALB/c mice were injected i.v. with 100 µg of U-Omp19 untreated or digested with proteinase K or 25 µg of *E. coli* LPS (Sigma) or with PBS alone. Twenty hours after immunization, mice were sacrificed and spleen cells from mice were removed and treated for 45 min at 37°C with 400 U/ml collagenase type IV and 50 mg/ml DNase I (Boehringer Mannheim) in RPMI 1640. After inhibition of collagenase with 6 mM EDTA and 0.5% fetal calf serum, a single spleen cell suspension was prepared and incubated with PE-anti-CD11c mAb (BD Pharmingen) and FITC conjugates anti-CD86, anti-CD40 and anti-CD80. After staining, cells were fixed and analyzed by flow cytometry using a FACS ARIA II (BD Biosciences). Data were analyzed using FlowJo software (Tree Star).

### Quantification of U-Omp19 in crude leaves of tobacco material

The U-Omp19 content in crude leaves of tobacco material was evaluated by a competitive ELISA based on a rabbit anti-U-Omp19 polyclonal serum and the recombinant purified *E. coli*-made U-Omp19 as standard.

Polystyrene plates (Immuno plate with MaxiSorp surface Nunclon; Nunc, Roskilde, Denmark) were coated with 0.5 µg/well of purified recombinant U-Omp19 in PBS. After 1 h of incubation at room temperature plates were washed 4 times in PBS with 0.05% Tween 20 (PBS-T), and blocked with 200 µl/well of 3% skim milk in PBS overnight at 4°C. A 1∶1 mix of the rabbit anti-Omp19 serum (1∶32000) with serial dilutions of U-Omp19 (standard curve) or the samples (soluble crude leaves material) were incubated at room temperature for 30 min and afterwards added to the blocked plates (50 µl/well) and incubated 1 h at room temperature. Each condition was carried out in triplicate. The serum was diluted in PBS-T containing 1.5% skim milk and the standard U-Omp19 and samples were diluted in PBS. Isotype-specific goat anti-rabbit horseradish peroxidase conjugate (Santa Cruz Biotechnology, Santa Cruz, CA) was added (50 µl/well) at an appropriate dilution. After 1 h of incubation at room temperature, plates were washed 4 times, and 50 µl/well of substrate solution (200 µM of o-phenylenediamine and 0.04% H_2_O_2_) were added to each well. After 20 min of incubation at room temperature, the enzyme reaction was stopped by addition H_2_SO_4_ and the absorbance was measured at 492 nm. The absorbance values were transformed using the following equation log_10_ (A/(A_max_-A) were A is the measured absorbance for a standard concentration or a sample and A_max_ is the absorbance measured when any soluble U-Omp19 was incubated with the rabbit serum. This transformation leaded to a lineal relation with log_10_C (C =  concentration of U-Omp19). This immunoassay was highly specific, sensitive, and suitable for U-Omp19 quantification. U-Omp19 concentrations determinable by ELISA ranged from 0.078 to 300 µg/ml.

### *In vivo* CD4^+^ T cell depletion or IL-17 neutralization

In some experiments immunized mice were depleted of CD4^+^ T cells prior to the challenge with virulent *Brucella* while other groups of mice received anti-IL-17A mAb (Biolegend) after the challenge to neutralize IL-17 activity. For the CD4^+^ T cell depletion, vaccinated mice were i.p. injected with 200 µg of purified GK1.5 (American Type Culture Collection) mAb, on days –15, –13, –8, –5, and –1 before the bacterial challenge. The efficacy of cell depletion was determined by flow cytometry analysis of splenocytes and was greater than 98% (not shown). On the other hand, other groups of mice received i.p. 100 µg of i) purified anti-IL-17A mAb clone TC11-18H10.1 (Biolegend) or ii) a nonspecific rat immunoglobulin G (IgG) purified mAb (isotype control) on days 1, 7, 13, 20 after the bacterial challenge.

### Statistical analysis

GraphPad Prism 4 software (GraphPad, San Diego, CA) was used for Statistical analysis and plotting. Student test or the one-way ANOVA test followed by the Dunnett multiple-comparison posttest was used to analyze the protection results. The antibody titers were compared using the nonparametric Mann-Whitney U test.

Cytokine production and DTH response data were analyzed using the Student t test. Not normally distributed data were logarithmically transformed before the statistical analysis, after the transformation all parameters followed a normal distribution. A *P* value <0.05 was taken as the level of significance.
